# Transcranial Direct Current Stimulation for Patients With Pharmacoresistant Epileptic Spasms: A Pilot Study

**DOI:** 10.3389/fneur.2019.00050

**Published:** 2019-02-05

**Authors:** Dongju Yang, Qiaoyi Du, Zhaoyang Huang, Liping Li, Zhang Zhang, Liping Zhang, Xin Zhao, Xuan Zhao, Ting Li, Yicong Lin, Yuping Wang

**Affiliations:** ^1^Department of Neurology, Xuanwu Hospital, Capital Medical University, Beijing, China; ^2^Beijing Key Laboratory of Neuromodulation, Beijing, China; ^3^Department of Pediatric, Xuanwu Hospital, Capital Medical University, Beijing, China; ^4^Center of Epilepsy, Beijing Institute for Brain Disorders, Capital Medical University, Beijing, China

**Keywords:** tDCS, epileptic spasms, seizures, epilepsy, infantile spasms, EEG

## Abstract

**Background:** Epileptic spasms (ES) is a severe seizure type and lack of adequate methods for controlling of clinical attacks. Previous studies have indicated that cathodal transcranial direct current stimulation (tDCS) reduces seizure frequency for patients with epilepsy. ES are proposed to have a focal cortical origin. We hypothesized that patients with ES exhibit hyperactive network hubs in the parietal lobe, and that cathodal tDCS targeting the bilateral parietal region can reduce seizure frequency in patients with pharmacoresistant ES.

**Materials and Methods:** The present study consisted of three basic phases: (a) a pre-treatment monitoring period for 14 days; (b) a consecutive 14-day treatment period during which patients were treated with 1 or 2 mA cathode tDCS for 40 min once per day; (c) and a follow-up period for at least 28 days. During the first 20 min of treatment, the cathode was placed over the right parietal lobe (P4) with the reference electrode over the contralateral supra-orbital area. In the second 20 min, the cathode was placed over the left parietal lobe (P3), with the reference electrode over the contralateral supra-orbital area. All patients received active tDCS treatment, and some patients underwent more than one treatment block. Patients maintained a seizure diary throughout the study. Antiepileptic drug therapy remained unchanged throughout the study. K-related samples Friedman tests and two-related samples tests were used to analyze data from all patients.

**Results:** Seven patients with pharmacoresistant ES were included, receiving a total of eighteen 14-day blocks of tDCS treatment. We observed a significant difference in seizure frequency at the second month (*p* = 0.028, unadjusted), as well as a trend toward decreased seizure frequency at the fourth month (*p* = 0.068, unadjusted) of the first follow-up, relative to baseline. Three of seven patients (42.9%) exhibited sustained seizure reduction, while one (14.3%) experienced a short-term reduction in seizure frequency following cathodal tDCS treatment. Treatment was well tolerated in all patients.

**Conclusions:** Repeated tDCS with the cathode placed over the bilateral parietal region is safe and may be effective for reducing seizure frequency in a subgroup of patients with pharmacoresistant ES.

## Introduction

Epileptic spasms (ES) typically manifest as sudden flexion, extension or mixed extension-flexion of predominantly proximal and truncal muscles and are usually more sustained than myoclonic movements but not as sustained as tonic seizures (1). ES constitute the largest single epilepsy subgroup in the infantile period (2). Most cases of ES occur within the first year [i.e., infantile spasms (IS)]. West syndrome, as a highly recognized subgroup of IS, is characterized by typical ES seizures, distinct electroencephalography (EEG) patterns of hypsarrhythmia, and psychomotor delay/arrest (3). ES can also appear after the first year [i.e., late-onset epileptic spasms (LOES)]. While LOES account for a small number of ES cases, research has gradually revealed that it is a distinct condition with heterogeneity. Patients with LOES seldom exhibit the classic EEG pattern of hypsarrhythmia and often present with psychomotor regression and seizures resistant to antiepileptic drugs (AEDs) (4).

Adrenocorticotropic hormone (ACTH) and vigabatrin are recommended for patients with IS (5). However, the side effects of ACTH treatment have been well documented, including irritability, cushingoid features, hypertension, and hypokalemia (6). Furthermore, the need for intramuscular (IM) administration of ACTH has restricted usage of the drug. Although vigabatrin has been approved by the Food and Drug Administration (FDA), it carries a black-box warning for potentially permanent visual impairment (7). As the responder rate for spasm cessation ranges from 29 to 76% after vigabatrin, corticosteroids, or ACTH therapy (5), it is important to identify alternative treatments for patients with pharmacoresistant ES.

According to the new operational classification of seizure types issued by the International League Against Epilepsy (ILAE), ES onset can be classified as focal, generalized, or unknown (8). Although the underlying pathogenesis of ES is not fully understood, some neuroimaging studies have indicated that ES is associated with focal cortical changes (9). In addition, focal cortical involvement and propagation shown in ictal invasive EEG, and favorable outcomes following removal of epileptogenic lesions were reported in patients with ES (9, 10). These findings support the notion that ES may have a focal cortical origin. Magnetoencephalography (MEG) studies have recorded ictal symptoms of ES, and the ictal equivalent current dipoles were reported to be clustered in the parietal region (11, 12). Functional magnetic resonance imaging (fMRI) studies have revealed that regional homogeneity (ReHo) values in the left precuneus and right superior frontal gyrus are associated with epilepsy duration in patients with IS (13). Therefore, we hypothesized that patients with ES exhibit hyperactive network hubs in the bilateral parietal regions.

Transcranial direct current stimulation (tDCS) is a painless, non-invasive brain stimulation technique that has been widely utilized in recent years (14). Cortical excitability can be modulated by low-intensity brain polarization. The primary effect of tDCS is a polarity-dependent shift of the resting cell membrane potential toward depolarization or hyperpolarization (15). While the mechanisms underlying the effects of tDCS are not completely understood, they may involve changes in calcium-dependent synaptic plasticity (16), as well as non-synaptic effects (17).

Anodal tDCS generally increases cortical excitability, while cathodal tDCS generally decreases cortical excitability (18). Thus, several studies have explored the therapeutic effect of cathodal tDCS among patients with epilepsy. Fregni et al. first reported that a single session of cathodal tDCS could reduce interictal epileptic discharges (IEDs), with a trend toward decreasing seizure frequency, in patients with malformations cortical of development (19). Auvichayapat et al. reported similar results among patients with childhood epilepsy (20). Yook et al. (21) and San-Juan et al. (22) first reported the antiepileptic efficacy of repeated tDCS in a few case studies. Tekturk et al. and San-Juan et al. demonstrated that three or five consecutive sessions of cathodal tDCS applied over the temporal region reduced seizure frequency among patients with mesial temporal lobe epilepsy (23, 24). Researchers have suggested that tDCS can modulate the activity of epileptogenic networks (23). Indeed, one previous study reported that five consecutive sessions of cathodal tDCS over the primary motor cortex reduces seizure frequency and IEDs in patients with Lennox–Gastaut syndrome (LGS) (25).

To date, no studies have evaluated the effect of cathodal tDCS among patients with ES. We hypothesized that patients with ES exhibit hyperactive network hubs in the parietal region, and that cathodal tDCS over the bilateral parietal regions can decrease hyperactivity in these areas, potentially allowing for seizure control.

## Materials and Methods

### Participant Recruitment

Study participants were recruited from epilepsy center of Capital Medical University Xuanwu Hospital, China, if they fulfilled the following inclusion criteria: (a) presence of epileptic spasms, confirmed by ictal video-EEG; (b) failure of more than two first-line AEDs to control seizures. Since some patients experienced more than one type of seizure, video-EEG was used to classify seizure types in accordance with 2017 ILAE criteria (8).

Exclusion criteria were as follows: (a) diagnosis of LGS, psychogenic seizures, or major psychiatric-neurological disorders other than epilepsy; or (b) drug addiction, pregnancy, skull defects, or implantation of other electrical medical devices.

Written informed consent for the study and publication of study data was obtained from all participants and their guardians before inclusion. The study conformed to the Declaration of Helsinki. This study was approved by the local ethics committee (Ethics Committee of Capital Medical University Xuanwu Hospital).

### Experimental Design

All patients received active tDCS treatment. Each treatment block consisted of 14 consecutive days of cathodal tDCS treatment, and all patients underwent at least one treatment block. The study consisted of three basic phases: (a) a 14-day pre-treatment monitoring period; (b) a 14-consecutive-day treatment period; (c) and a follow-up period of at least 28 days. No changes were made to AED treatment throughout the study.

### tDCS

Cathodal tDCS was applied using a saline-soaked pair of surface sponge electrodes (20 cm^2^) and delivered through a specially designed, constant-current stimulator (Yunshen tech, China). Patients received 40 min of stimulation per day during each treatment session. In the first 20 min, the cathodal electrode was placed over P4 (in accordance with the international standardized 10–20 system for electrode placement), with a constant current of 1 or 2 mA. The reference electrode was placed over the left supra-orbital area. In the second 20 min, the cathodal electrode was placed over P3, while the reference electrode was placed over the right supra-orbital area.

**Figure 1:**
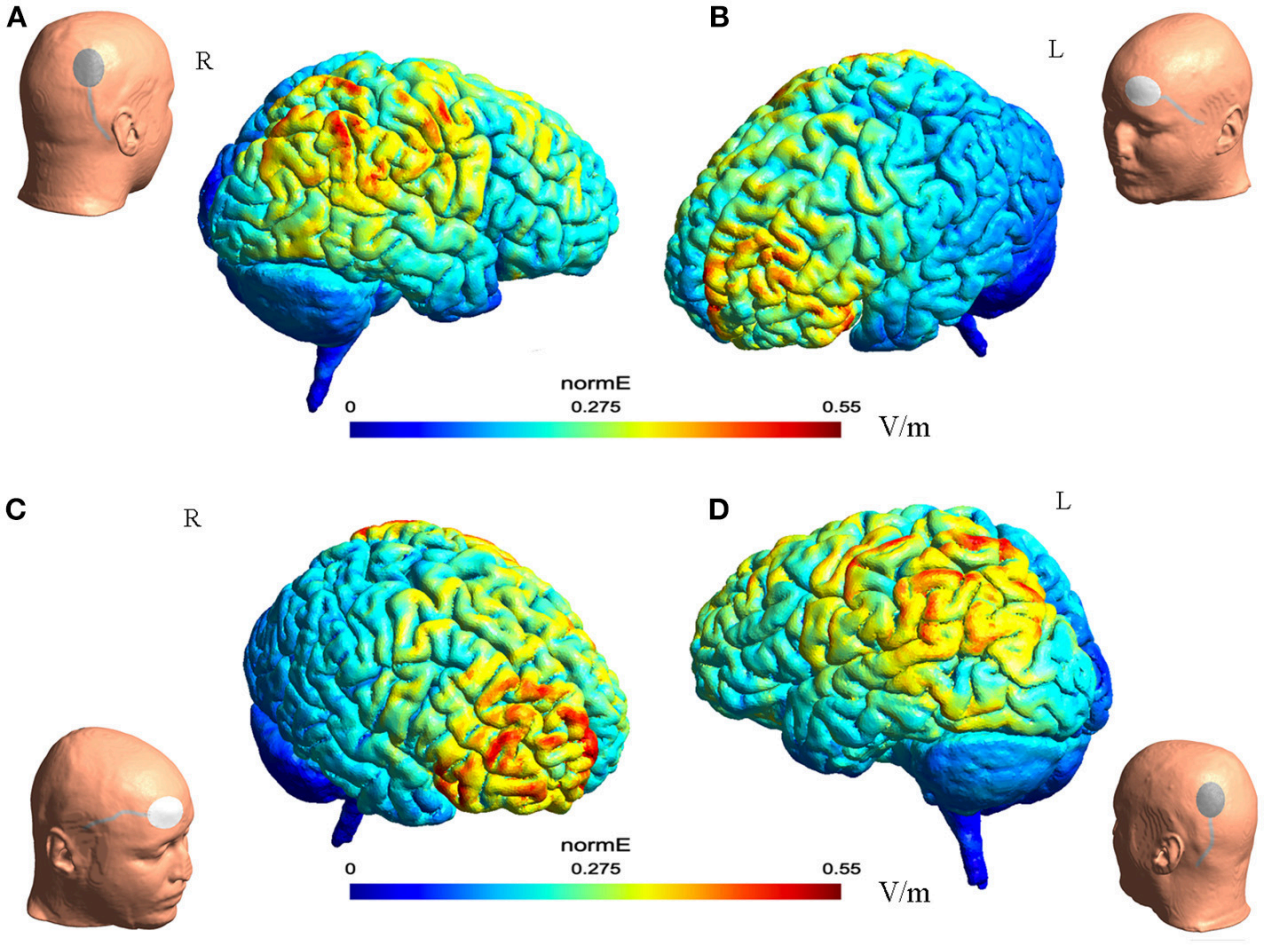
The illustration of electrode montage and estimated electric field for tDCS. The gray line indicated the cable feeding the tDCS electrodes. Together, **(A,B)** illustrate the montage for cathode-P4 and anode-left supra-orbital. **(A)** (Left) the illustration of cathode on P4 on a model head. (Right) the estimated electric field in the brain from the lateral view. **(B)** (Left) the estimated electric field in the brain from the lateral view. (Right) the illustration of anode on left supra-orbital area on a model head. **(C,D)** Illustrated together the montage of cathode-P3 and anode- right supra-orbital.

The electric field distribution in the brain Figure 1 was estimated using SimNIBS 2.1.1 software (26). The estimation was generated based on the template head model included in the software package, and the conductivity values for different biological tissue were as follows: SimNIBS default values, scalp (s = 0.465 S/m), bone (s = 0.010 S/m), cerebrospinal fluid (s = 1.654 S/m), gray matter (s = 0.275 S/m), and white matter (s = 0.126 S/m) (26).

### Outcome Measures and Statistical Analysis

Seizure frequency as recorded by the patients' caregivers was regarded as the main outcome measure. Since ES appeared in clusters in all patients, caregivers were asked to record every episode in each cluster.

Mean seizure frequency was calculated for different time periods. Seizure reduction was calculated using the following formula:

$$\frac{\left(seizure\;frequency\;of\;baseline-seizure\;frequency\;of\;certain\;period\right)}{seizure\;frequency\;of\;baseline}*100\%$$

An over 50% reduction in seizure frequency compared to baseline was defined as a positive response to tDCS treatment.

Statistical analysis was performed using SPSS V. 20. Non-parametric Wilcoxon signed-rank tests were used due to the small sample size. K-related samples Friedman tests were used to analyze seizure frequency at baseline, during the treatment and follow-up periods. Two-related samples tests were used for an exploratory analysis, in which seizure frequency in the treatment and follow-up periods was separately compared to seizure frequency at baseline. *P* < 0.05 (two-tailed) were considered statistically significant.

## Results

### Case Descriptions

#### Patient 1

**Figure 2:**
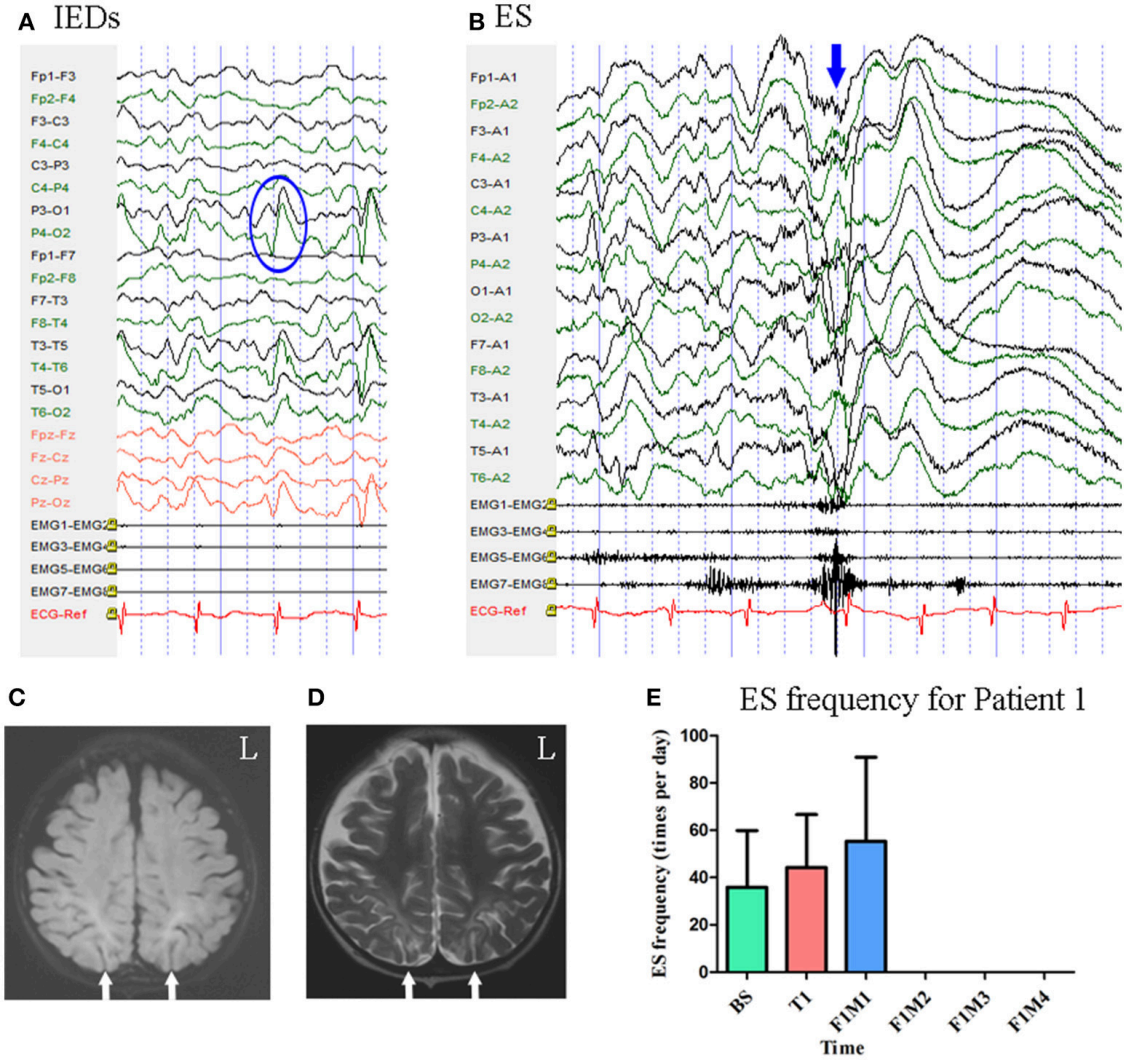
The EEG, MRI, and seizure frequency for Patient 1. **(A)** Interictal EEG for Patient 1 showed IEDs on bilateral parietal-occipital regions (blue circle). **(B)** Ictal ES EEG for Patient 1 showed generalized high-voltage slow waves (blue arrow) followed by a diffuse electro-decrement with superimposed fast activity. **(C,D)** MRI scan showed bilateral parietal-occipital region encephalomalacia (white arrow). **(C)** Flair MRI; **(D)** T2 MRI. **(E)** Seizure frequency of ES for Patient 1. The patient was seizure free for three months. BS, baseline; ES, epileptic spasm; F1M1-F1M4, the first to the fourth month of the follow-up; IEDs, interictal discharges; L, left; T1, treatment 1.

Patient 1 was a 2-year-old girl with a history of perinatal hypoglycemia and a 1.5-year history of ES. No other types of seizure were noted. MRI revealed bilateral parietal–occipital encephalomalacia. Interictal scalp EEG revealed sharp waves mainly over the bilateral parietal–occipital regions Figure 2. Video-EEG captured 40 episodes of ES, which presented as blinking accompanied by rapid jittering throughout the body, with ictal EEG showing generalized high-voltage slow waves followed by a diffuse electro-decrement with superimposed fast activity Figure 2 [i.e., typical ictal EEG pattern for ES (4)].

Mean seizure frequency during the baseline period was 35.90 ± 23.95 times per day, in spite of taking clonazepam, sodium valproate, lamotrigine, and topiramate. She underwent one block of tDCS treatment at 1 mA. A current intensity of 1 mA was used due to her age, however all other tDCS parameters remained the same as mentioned above. During the treatment period and the first month of follow-up, mean seizure frequency was 44.23 ± 22.36 and 55.33 ± 35.56 times per day, respectively. She remained seizure free throughout the next 3 months of follow-up. Mean seizure frequency for Patient 1, who was classified as a positive responder, is shown in Figure 2.

#### Patient 2

**Figure 3:**
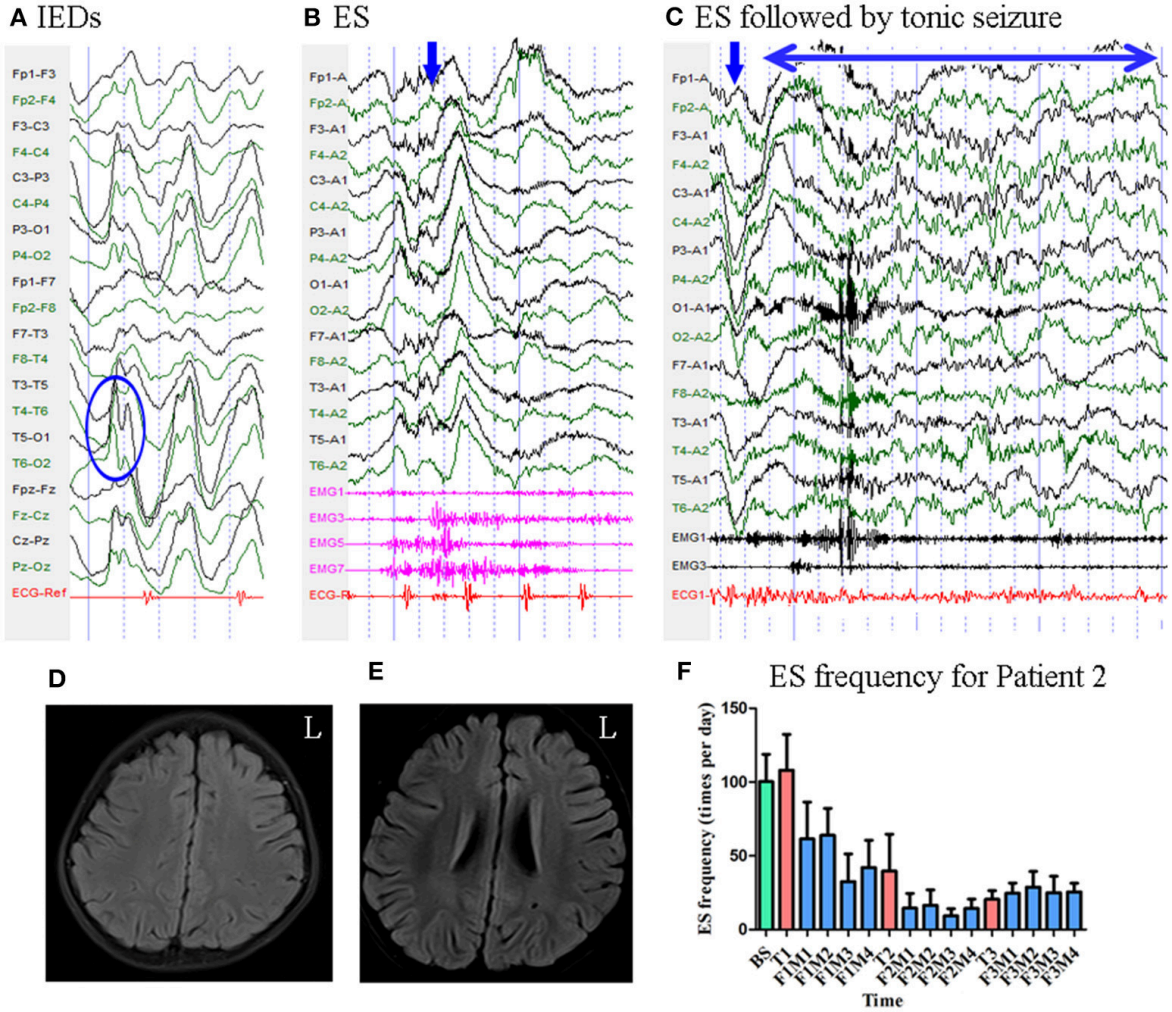
The EEG, MRI and seizure frequency for Patient 2. **(A)** Interictal EEG for Patient 2 showed sharp and slow wave complexes mainly on bilateral parietal-occipital-posterior temporal regions (blue circle). **(B)** Ictal ES EEG for Patient 2 showed typical ictal EEG pattern of ES (blue arrow marks the high amplitude slow wave). **(C)** Ictal ES -TS EEG for Patient 2 showed a constant spike rhythm following typical ES pattern (blue arrow indicated the slow wave; double sided blue arrow indicated the time course of the spike rhythm). **(D,E)** MRI (flair) scan showed no evidence of lesion. **(F)** Seizure frequency of ES for Patient 2. The patient had more than 50% seizure reduction for 12 months. BS, baseline; ES, epileptic spasm; ES-TS, ES followed by a tonic seizure; F1M1-F1M4, the first to the fourth month of the first follow-up; F2M1-F2M4, the first to the fourth month of the second follow-up; F3M1-F3M4, the first to the fourth month of the third follow-up; IEDs, interictal discharges; L, left; T1, treatment 1; T2, treatment 2; T3, treatment 3.

Patient 2 was a 3-year-old girl with a 6-month history of ES. MRI revealed no evidence of lesions. Scalp EEG revealed sharp and slow wave complexes mainly over the bilateral parietal–occipital–posterior temporal regions Figure 3. Two types of seizure were captured by ictal video-EEG: (a) 77 episodes of ES, which presented as nodding accompanied by rapid lifting of the upper limbs, with a typical ictal EEG pattern for ES Figure 3; (b) one episode of ES followed by a tonic seizure, which presented as sudden nodding, lifting, and stiffening of the upper limbs for several seconds, with EEG showing a spike rhythm following a typical ES pattern Figure 3.

Mean seizure frequency at baseline was 100.33 ± 18.44 times per day. She was taking sodium valproate, clonazepam, topiramate, lamotrigine, and levetiracetam. The patient underwent three blocks of tDCS treatment at 2 mA. Mean seizure frequency during the first, second, and third months of follow-up was 52.09 ± 24.73, 14.17 ± 8.77, and 26.12 ± 9.23 times per day, respectively Figure 3. As Patient 2 experienced a more than 50% reduction in seizure frequency for 12 months, she was defined as a positive responder.

#### Patient 3

**Figure 4:**
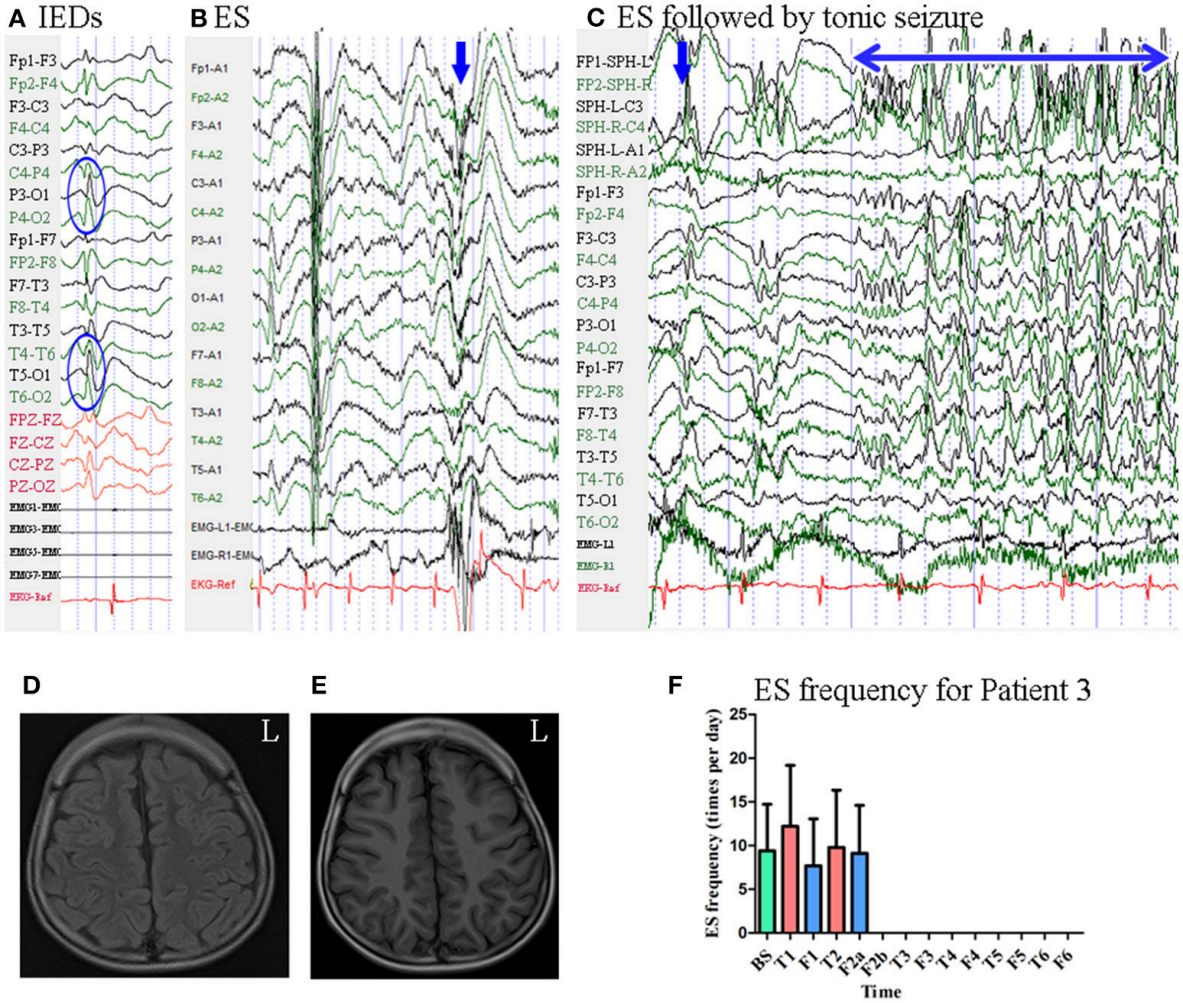
The EEG, MRI, and seizure frequency for Patient 3. **(A)** Interictal EEG for Patient 3 showed sharp waves mainly on bilateral parietal-occipital-posterior temporal regions (blue circle). **(B)** Ictal ES EEG for Patient 3 showed typical ictal EEG pattern of ES (blue arrow marks the high amplitude slow wave). **(C)** Ictal ES-TS EEG for Patient 3 showed a constant spike rhythm following typical ES pattern (blue arrow indicated slow wave; double sided blue arrow indicated the time course of spike rhythm). **(D,E)** MRI scan showed no evidence of lesion; **(D)**, flair MRI; **(E)**, T1 MRI. **(F)**. Seizure frequency of ES for Patient 3. The patient was seizure free since the 20th day of second follow-up for 6 months. BS, baseline; ES, epileptic spasm; ES-TS, ES followed by a tonic seizure; F1, the first follow-up; F2a, the first 19 days of the second follow-up; F2b, the remain 9 days of the second follow-up; F3, the third follow-up; F4, the fourth follow-up; F5, the fifth follow-up; F6, the sixth follow-up; IEDs, interictal discharges; L, left; T1, treatment 1; T2, treatment 2; T3, treatment 3; T4, treatment 4; T5, treatment 5; T6, treatment 6.

Patient 3 was a 7-year-old boy with a 3-year history of ES. MRI revealed no evidence of lesions. Scalp EEG revealed sharp waves mainly over the bilateral parietal–occipital–posterior temporal regions Figure 4. Two types of seizure were captured by ictal video-EEG: (a) 14 episodes of ES, which presented as nodding, blinking, deviation of the right corner of the mouth, accompanied by rapid lifting of the upper limbs, with a typical ictal EEG pattern for ES Figure 4; (b) one episode of ES followed by a tonic seizure, which presented as sudden nodding, lifting, and stiffening of the upper limbs for several seconds, with EEG showing a spike rhythm following a typical ES pattern Figure 4.

Mean seizure frequency at baseline was 9.41 ± 5.30 times per day. He was taking sodium valproate, clonazepam, and topiramate. The patient underwent six blocks of tDCS treatment at 2 mA. Seizure frequency at the first follow-up was 7.68 ± 5.38 times per day. During the first 19 days of the second follow-up, seizure frequency was 9.11 ± 5.49 times per day. Beginning the 20th day of the second follow-up, he remained seizure-free for 6 months. Thus, he was identified as a positive responder. Mean seizure frequency for Patient 3 is shown in Figure 4.

#### Patient 4

**Figure 5:**
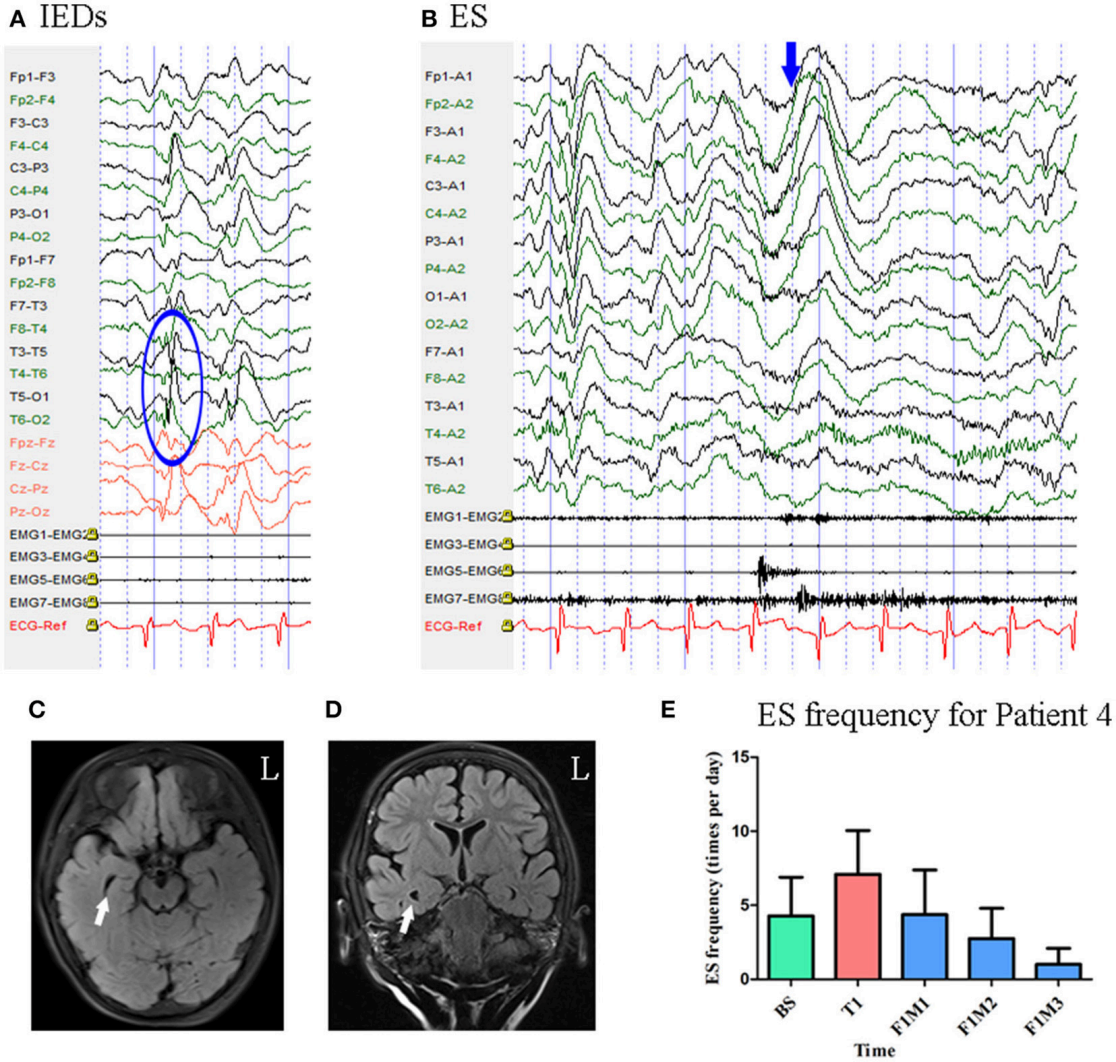
The EEG, MRI and seizure frequency for Patient 4. **(A)** Interictal EEG for Patient 4 showed IEDs on posterior temporal- parietal regions with higher amplitude on left side (blue circle). **(B)** Ictal ES EEG for Patient 4 showed typical ictal EEG pattern of ES (blue arrow marks the high amplitude slow wave). **(C,D)** MRI (flair) scan showed enlargement of the right temporal horn of lateral ventricle (white arrow). **(E)**. Seizure frequency of ES for Patient 4. The seizure frequency reduction reached 76.64% at the third month of follow-up. BS, baseline; ES, epileptic spasm; F1M1-F1M3, the first to the third month of the first follow-up; IEDs, interictal discharges; L, left; T1, treatment 1.

Patient 4 was a 3-year-old girl with a 1-year history of ES. MRI revealed enlargement of the right temporal horn of the lateral ventricle. Scalp EEG revealed sharp and wave complexes mainly over the posterior temporal–parietal regions, with higher amplitude on the left side electrodes Figure 5. One type of seizure was captured by ictal video-EEG: 146 episodes of ES, which presented as nodding and lifting of the bilateral upper limbs, with typical ictal EEG patterns for ES Figure 5.

Mean seizure frequency at baseline was 4.28 ± 2.61 times per day. She was taking sodium valproate, nitrazepam, levetiracetam, and topiramate. The patient underwent one block of tDCS treatment at 2 mA. Her seizure frequency began to decrease during the second month of the follow-up period and reached a 76.64% reduction by the third month Figure 5. Thus, she was identified as a positive responder.

#### Patient 5

**Figure 6:**
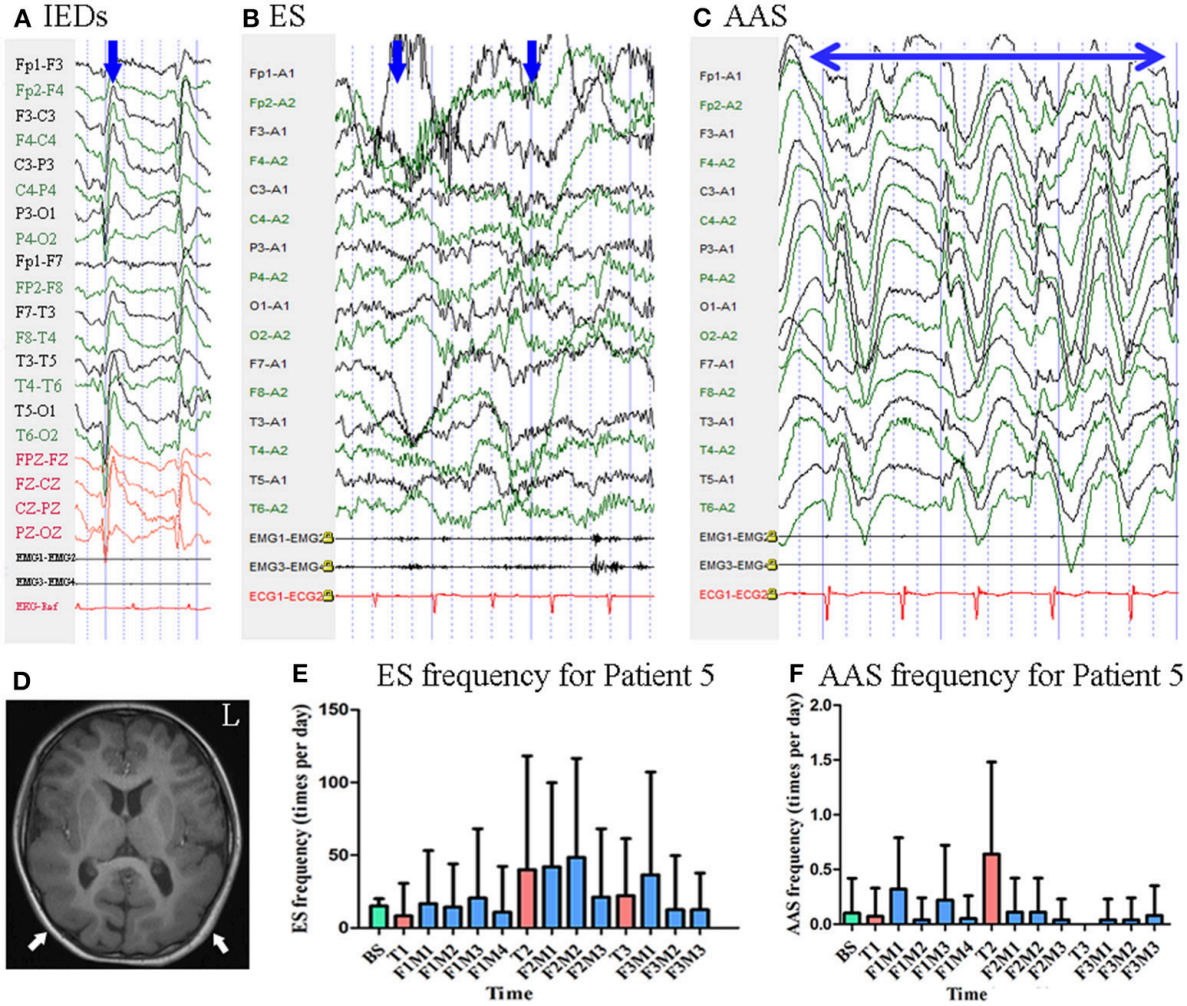
The EEG, MRI and seizure frequency for Patient 5. **(A)** Interictal EEG for Patient 5 showed IEDs on bilateral central-parietal-posterior temporal regions (blue arrow). **(B)** Ictal ES EEG for Patient 5 showed typical ictal EEG pattern of ES (blue arrow indicated two ictal ES episodes). **(C)** Ictal atypical absence seizure EEG for Patient 5 showed synchronous high amplitude 1.5 Hz sharp and wave complexes (the double sided blue arrow indicated the time course of sharp and wave complex rhythm). **(D)** MRI (T1) scan showed pachygyria in the cortex (white arrow). **(E)** Seizure frequency of ES for Patient 5. **(F)** Seizure frequency of AAS for Patient 5. AAS, atypical absence seizure; BS, baseline; ES, epileptic spasm; F1M1-F1M4, the first to the fourth month of the first follow-up; F2M1-F2M3, the first to the third month of the second follow-up; F3M1-F3M3, the first to the third month of the third follow-up; IEDs, interictal discharges; L, left; T1, treatment 1; T2, treatment 2; T3, treatment 3.

Patient 5 was a 9-year-old girl with a 7-year history of ES. MRI revealed pachygyria in the cortex. Scalp EEG revealed spike and wave complexes mainly over the bilateral central–parietal–posterior temporal regions Figure 6. Two types of seizure were captured by ictal video-EEG: (a) 15 episodes of ES, which presented as nodding and flexion of the trunk, with typical ictal EEG patterns for ES; (b) four episodes of atypical absence seizures, which presented as loss of awareness, with EEG showing generalized high-amplitude (1.5 Hz) sharp and slow wave complexes Figure 6.

Mean seizure frequency for ES at baseline was 15.00 ± 5.00 times per day. She was taking sodium valproate, clonazepam, and lamotrigine. The patient underwent three blocks of tDCS treatment at 2 mA. Mean seizure frequency for ES during the first, second, and third months of follow-up was 15.76 ± 35.91, 38.39 ± 59.57, and 21.02 ± 49.78 times per day, respectively Figure 6 Mean seizure frequency for atypical absence seizures at baseline was 0.10 ± 0.32 times per day. Mean seizure frequency for atypical absence seizures during the first, second, and third months of follow-up was 0.16 ± 0.40, 0.08 ± 0.27, 0.05 ± 0.22 times per day, respectively Figure 6. As Patient 5 underwent three tDCS blocks without achieving a 50% reduction in seizure frequency, she was not identified as a positive responder.

#### Patient 6

**Figure 7:**
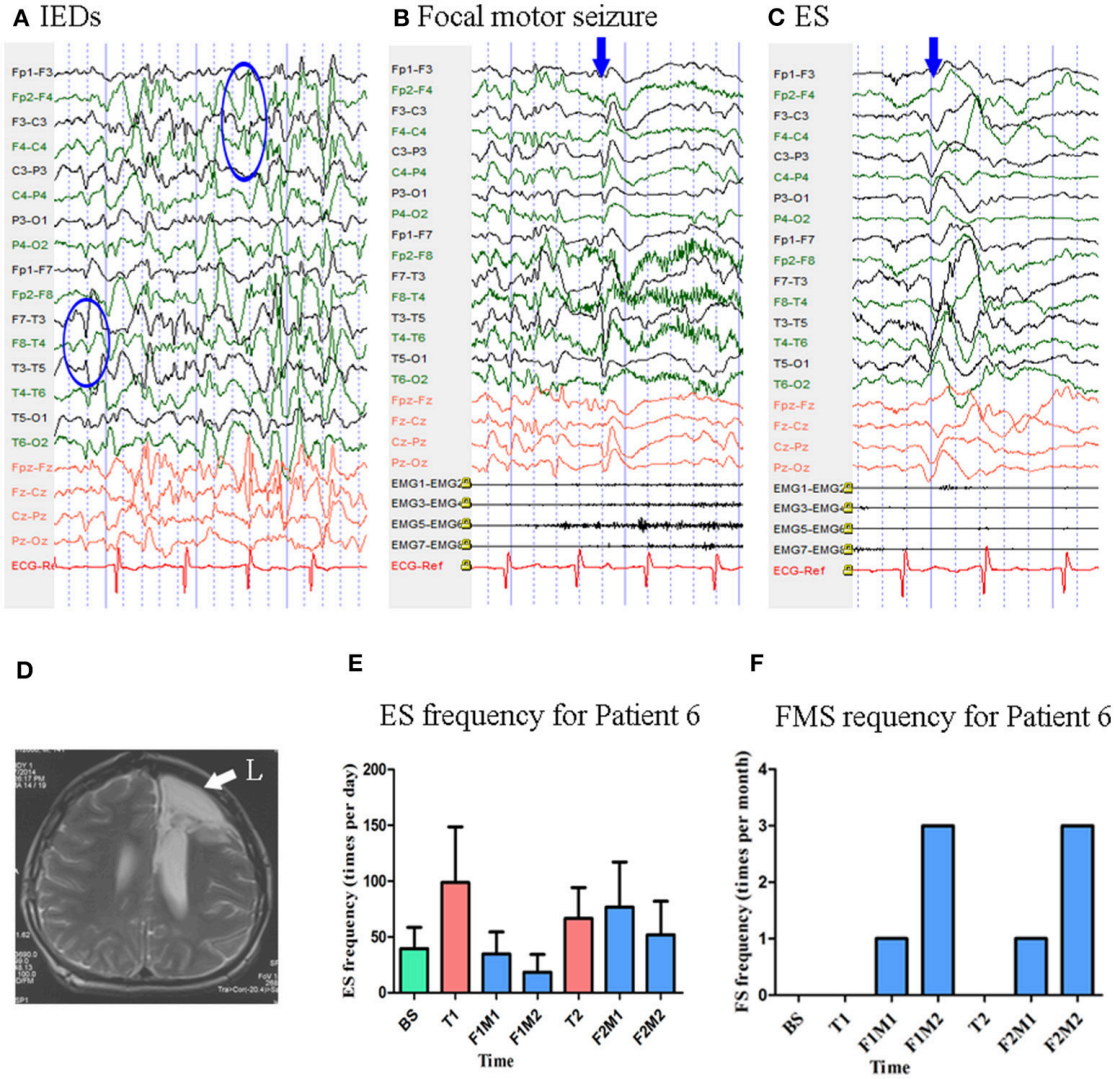
The EEG, MRI and seizure frequency for Patient 6. **(A)** Interictal EEG for Patient 6 showed IEDs on right frontal and left temporal electrodes (blue circles). **(B)** Ictal focal motor seizure EEG for Patient 6 showed low amplitude of fast rhythm on the right frontal-temporal electrodes, the blue arrow indicated the onset of symptoms. **(C)** Ictal ES EEG for Patient 6 showed typical ictal EEG pattern of ES (blue arrow indicated the high amplitude slow wave). **(D)** MRI (T2) showed post-operative change in left frontal lobe (white arrow). **(E)** Seizure frequency of ES for Patient 6. **(F)** Seizure frequency of FS for Patient 6. BS, baseline; ES, epileptic spasm; F1M1-F1M2, the first to the second month of the first follow-up; F2M1-F2M2, the first to the second month of the second follow-up; FMS, focal motor seizure; IEDs, interictal discharges; L, left; T1, treatment 1; T2, treatment 2.

At the time of enrollment, Patient 6 was a 15-year-old boy who had been experiencing ES and focal motor seizures since the age of 1 year. He underwent left frontal lobectomy at the age of 14, although no significant changes in seizure frequency were observed following surgery. MRI revealed post-operative changes in the left frontal lobe and abnormal signals in the posterior horn of the bilateral ventricles. Scalp EEG revealed sharp waves over the right frontal and left temporal regions (Figure 7). Two types of seizure were captured by ictal video-EEG: (a) 71 episodes of ES, which presented as nodding toward the left or right side accompanied by blinking, with typical ictal EEG patterns for ES; (b) one episode of focal motor seizures, which presented as dystonia and clonus of the left upper limb followed by trunk stiffness, with simultaneous EEG showing low-amplitude fast activity over the right frontal–temporal region (Figure 7).

Mean seizure frequency for ES at baseline was 39.60 ± 19.06 times per day. He was taking sodium valproate, zonisamide, and lamotrigine. The patient underwent two blocks of tDCS treatment at 2 mA. Mean ES frequency was 29.19 ± 20.39 and 69.23 ± 38.83 times per day during the first and second follow-up, respectively Figure 7. No focal motor seizures were observed during the 14-day baseline period. Mean focal motor seizure frequency was 2.00 times per 28-days during both the first and second follow-up Figure 7. As Patient 6 underwent two tDCS blocks without achieving a 50% reduction in seizure frequency, he was not identified as a positive responder.

#### Patient 7

**Figure 8:**
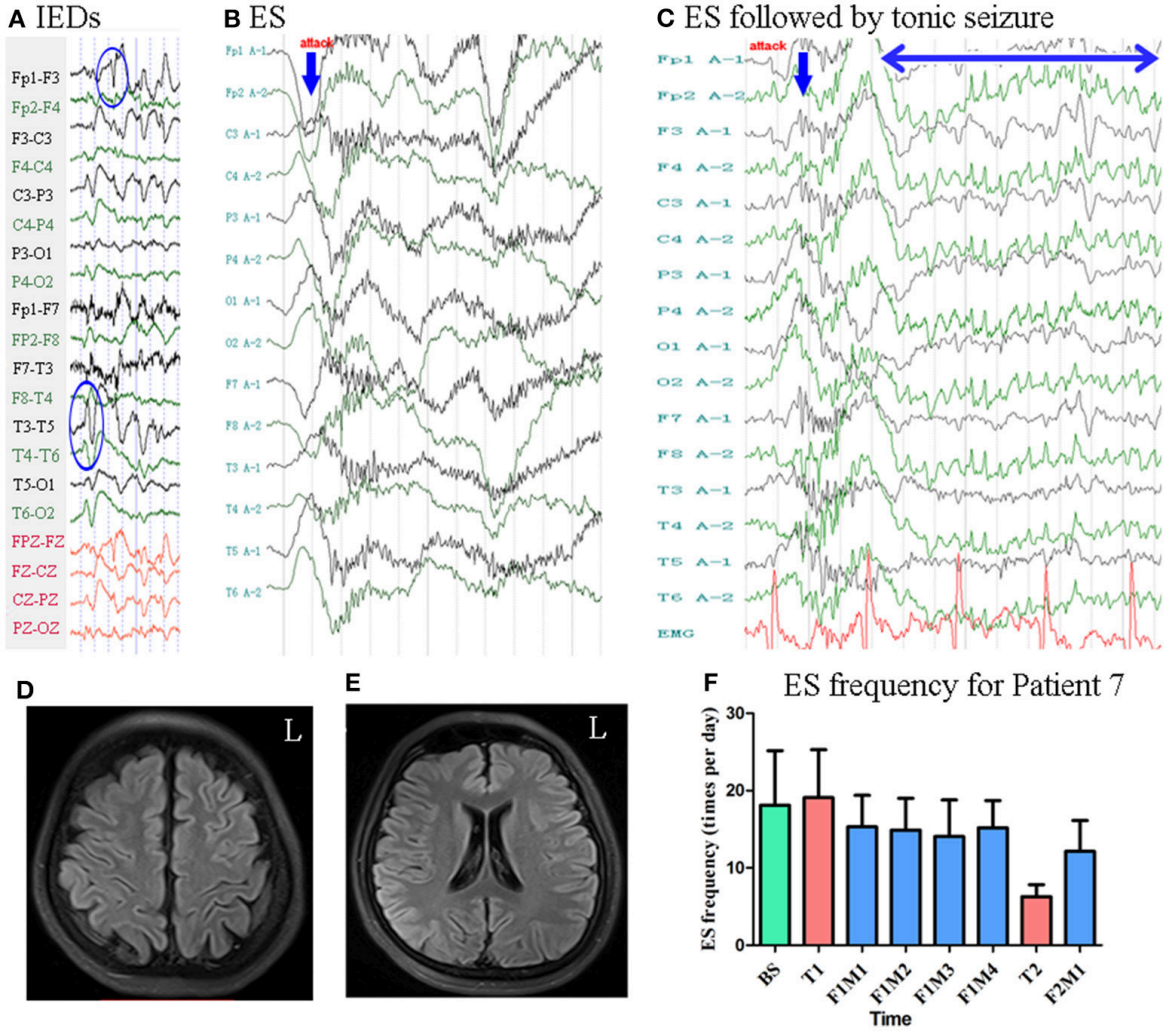
The EEG, MRI and seizure frequency for Patient 7. **(A)** Interictal EEG for Patient 7 showed IEDs on bilateral frontal-temporal regions (blue circles). **(B)** Ictal ES EEG for Patient 7 showed typical ictal EEG pattern of ES (blue arrow indicated the high amplitude slow wave). **(C)** Ictal ES-TS EEG for Patient 7 showed a constant spike rhythm following typical ES pattern (blue arrow indicated high amplitude slow wave; double sided blue arrow indicated the time course of spike rhythm). **(D,E)** MRI (flair) showed no lesion. **(F)** Seizure frequency of ES for Patient 7. BS, baseline; ES, epileptic spasm; ES-TS, ES followed by a tonic seizure; F1M1-F1M4, the first to the fourth month of the first follow-up; F2M1, the first month of the second follow-up; IEDs, interictal discharges; L, left; T1, treatment 1; T2, treatment 2.

Patient 7 was a 25-year-old woman with an 8-year history of ES. MRI revealed no evidence of lesions at the time of enrollment. She had a history of IS at the age of 5 months, at which time MRI revealed subdural effusion. Following drill drainage, she remained seizure-free until the age of 17 years. Scalp EEG revealed sharp waves and complexes mainly over the bilateral frontal–temporal regions Figure 8. Two types of seizure were captured by ictal video-EEG: (a) seven episodes of ES, which presented as slight nodding, with typical ictal EEG patterns for ES; (b) one episode of ES followed by a tonic seizure, which presented as sudden, slight nodding and stiffness of the neck for several seconds, with EEG showing spike rhythms following a typical ES pattern Figure 8.

Mean seizure frequency at baseline was 18.10 ± 7.05 times per day. She was taking sodium valproate, clonazepam, zonisamide, and lamotrigine. The patient underwent two blocks of tDCS treatment at 2 mA. Mean seizure frequency was 14.81 ± 4.20 and 12.15 ± 4.00 times per day during the first and second follow-up, respectively Figure 8. As Patient 7 underwent two tDCS blocks without achieving a 50% reduction in seizure frequency, she was not identified as a positive responder.

### Summary of Cases

Seven patients were included, undergoing a total of 18 tDCS treatment blocks (14 days each). Two patients underwent only one treatment block, two underwent two blocks, two underwent three blocks, and one underwent six blocks. In all patients, tDCS was well tolerated without severe side effects, although some patients reported experiencing an itching sensation during the treatment period. Three patients (42.9%) (Patients 1–3) experienced long-lasting decreases in seizure frequency, while one patient (14.3%, Patient 4) experiencing a short-term decrease in seizure frequency, following cathodal tDCS treatment, as shown in Table 1. Among all patients who responded well to tDCS, seizure reduction occurred during follow-up rather than the treatment period. In Patients 2 and 3, seizure frequency continued to decrease as the number of treatment blocks increased.

**Table 1:** Summary for all cases.

**Case**	**G**	**A**	**Du**	**MRI**	**IEDs**	**TB**	**mA**	**ST**	**Re**
1	F	2Y	1Y6Mo	bi PO encephalomalacia	bi P-O	1	1	ES 100%	Yes
2	F	3Y	6Mo	no lesion	bi P-O-pT	3	2	ES 98.72%	Yes
								ES-TS 1.28%	
3	M	7Y	3Y	no lesion	bi P-O-pT	6	2	ES 93.33%	Yes
								ES-TS 6.67%	
4	F	3Y	1Y	enlargement of the right temporal horn of lateral ventricle	L pT-P	1	2	ES 100%	Yes
5	F	9Y	7Y	pachygyria	bi C-P-pT	3	2	ES 78.95%	No
								AAS 21.05%	–
6	M	15Y	14Y	post-operative change in left F	R Fo, L T	2	2	ES 98.61%	No
								FMS 1.39%	–
7	F	25Y	8Y	no lesion	bi Fo-T	2	2	ES 87.50%	No
								ES-TS 12.5%	

*A. age; AAS, atypical absence seizure; bi, bilateral; C, central lobe; Du, Disease duration; ES, epileptic spasms; ES-TS, ES followed by tonic seizure; F, female; Fo, frontal lobe; FMS, focal motor seizure; G, gender; IEDs, interictal discharges; L, left; M, male; Mo, month; O, occipital lobe; P, parietal lobe; pT, posterior temporal lobe; R, right; Re, whether was a positive responder; ST, seizure types; T, temporal lobe; TB, treatment blocks; Y, year-old*.

### Statistical Analysis

**Figure 9:**
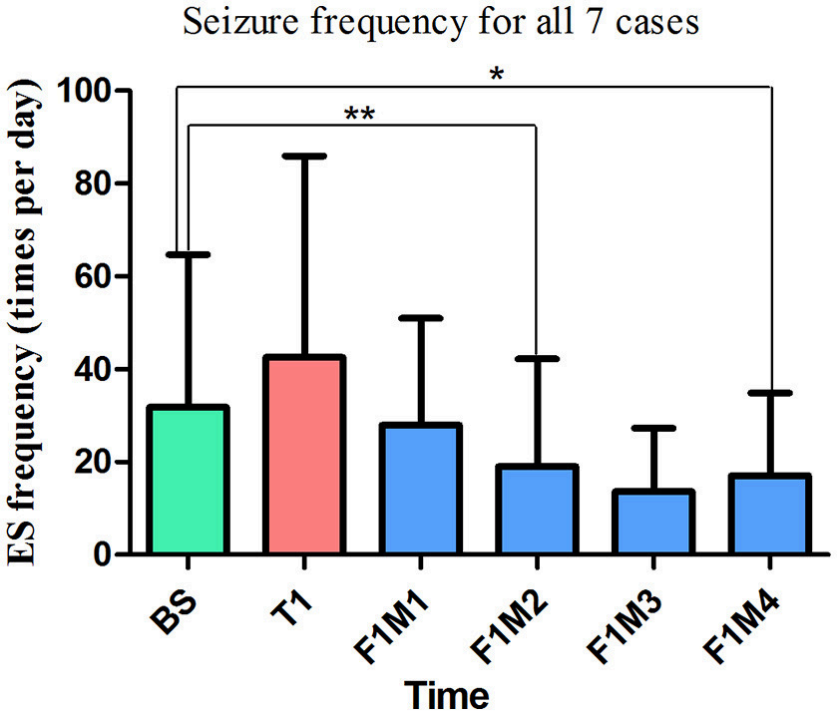
The mean seizure frequency of ES for all patients. In the exploratory analysis, we observed a significant reduction in seizure frequency at the second month of the first follow-up period (F1M2, *p* = 0.028, unadjusted, *n* = 6), as well as a trend toward decreased seizure frequency at the fourth month (F1M4, *p* = 0.068, unadjusted, *n* = 4) of the first follow-up period. Relative to baseline, mean reductions in seizure frequency at T1, F1M1, F1M2, F1M3, and F1M4 were −33.71, 12.14, 40.13, 57.14, and 46.51%, respectively. ^**^Significant difference in seizure frequency, (*p* = 0.028, unadjusted). ^*^Marginally significant difference in seizure frequency (*p* = 0.068, unadjusted). BS, baseline; ES, epileptic spasm; F1M1-F1M4, the first to the fourth month of the first follow-up; T1, treatment 1.

Only the first treatment and the first follow-up were completed by all seven patients, so only data from these periods were included for statistical analysis. Thus, there were six time points included: baseline (BS, *n* = 7), treatment 1 (T1, *n* = 7), the first month of follow-up (F1M1, *n* = 7), the second month of follow-up (F1M2, *n* = 6), the third month of follow-up (F1M3, *n* = 5), and the fourth month of follow-up (F1M4, *n* = 4). K-related samples Friedman tests revealed no significant differences in seizure frequency among these six time points (*p* = 0.128). In an exploratory analysis, we then separately compared seizure frequency at the five time points (T1-F1M4) with seizure frequency at baseline using two-related samples tests. We observed a significant difference in seizure frequency at the second month (F1M2, *p* = 0.028, unadjusted) of the follow-up period, when compared with the baseline. We also observed a trend toward seizure reduction at the fourth month (F1M4, *p* = 0.068, unadjusted) of the follow-up period, when compared with the baseline (Figure 9). *P*-values were not adjusted in the exploratory analysis (27, 28). Relative to baseline, mean reductions in seizure frequency at T1, F1M1, F1M2, F1M3, and F1M4 were −33.71, 12.14, 40.13, 57.14, and 46.51%, respectively.

## Discussion

In the present preliminary study, we evaluated the efficacy of tDCS in the treatment of pharmacoresistant ES. A significant reduction in seizure frequency at the second month (*p* = 0.028, unadjusted), as well as a trend toward reduction in seizure frequency at the fourth month (*p* = 0.068, unadjusted) of the follow-up was revealed following the first block of tDCS treatment for all patients. And among the seven included patients with ES, four experienced a <50% reduction in seizure frequency. This effect lasted for 3 months in Patient 1, 12 months in Patient 2, 6 months in Patient 3, and 1 month in Patient 4, influenced by the number of treatment blocks patient received.

### Potential Hubs in the Epileptic Network of ES

The pathophysiology of ES remains to be fully elucidated. While the earliest studies suggested that brainstem dysfunction can trigger ES (29), more recent studies indicate that ES may have a focal origin. Some studies have reported that ES can be triggered by focal seizures (30). In accordance with this result, Patient 6 of the present study had co-occurring motor seizures. Some neuroimaging studies have also reported that ES is associated with focal cortical changes. In one study that reviewed data from 80 patients with ES who underwent operations, 96.3% of patients exhibited MRI abnormalities, and 61.3% were classified as Engel class I (9). In our study, three of 7 patients had MRI abnormalities. The rate of patients exhibiting MRI abnormalities is lower in our study, because our patients were not all surgical patients. Another study reported that, among 65 surgical patients with ES, 92% exhibited lateralizing/localizing findings on positron emission tomography (PET) scans (30). Complete resection of the seizure onset zone (SOZ) (9), as well as lesions on MRI with EEG concordance (30), have been reported to be associated with favorable surgical outcomes. These findings suggest the involvement of cortex in ES.

We hypothesized that the bilateral parietal regions represent hubs in a hyperactive epileptic network in patients with ES. Thus, in accordance with previous findings, we selected P3 and P4 as treatment targets. A similar strategy has been adopted in tDCS treatment for patients with LGS. Auvichayapat et al. reported that tDCS targeting the primary motor cortex is effective for controlling seizures in patients with LGS (25). This finding is consistent with our results, suggesting that modulating potential network hubs through tDCS can aid in the treatment of epilepsy. In one study, the authors analyzed both generalized ES (affecting all four extremities) and focal ES (involving the legs only) recorded by MEG, reporting that both types of ES originated from the same region (right parietal region) but with different cortical spread patterns (11). Another study also recorded ictal MEG signals in patients with ES, and the ictal equivalent current dipoles were reported to be scattered in the right parietal region (12). IEDs were distributed locally in all cases of the present study Table 1. In our study, IEDs were located posteriorly in four patients (Patients 1–4), and anteriorly in three (Patients 5–7) patients, consistent with the finding that focal EEG abnormalities may be located anteriorly in some cases and posteriorly in others for patients with ES (4). Moreover, patients with posteriorly located IEDs (Patients 1–4) exhibited good responses to tDCS treatment, while those with anteriorly located IEDs did not (Patients 5–7).

### Characteristics of Good Responders

Good responders exhibited some common characteristics with regard to IEDs location, seizure types, age at tDCS treatment and disease duration prior to tDCS treatment. All good responders had IEDs mainly over the posterior regions, overlapping with the site of the cathodal electrode. In previous studies, EEG abnormalities were used to determine the placement of the cathode electrode, effectively controlling IEDs or seizures in patients with malformations cortical of development (19, 31), and patients with focal epilepsy with diverse etiologies, including generalized brain atrophy (20) and hippocampal sclerosis (24). The cathodal electrode was placed over the most active IED area, which was defined as the zone with the highest discharge amplitude and/or frequency in these studies. Thus, Patients 1–4 may have responded well to our treatment due to substantial overlap between tDCS targets and the active IED area. In contrast, the active IED area was located more anteriorly in non-responders (Patients 5–7), relatively farther from the site of the cathodal electrode.

Seizure types were also similar in good responders (Patients 1–4), who experienced either ES only or ES followed by tonic seizures. In contrast, atypical absence seizures and focal motor seizures were observed in Patients 5 and 6, respectively. Different seizure types may differ greatly with regard to symptoms, ictal EEG patterns, and mechanisms of cortical activity. Ronzano et al. reported that the occurrence of ES as the first seizure, at epilepsy onset, is significantly associated with a cryptogenic etiology, whereas onset with other types of seizures is associated with a symptomatic etiology (4). Epileptic networks may differ between patients with ES only (good responders in our study) and those with multiple seizure types (non-responders in our study). Taken together, our limited data suggest that tDCS can be effective in patients with ES, but not in patients with multiple seizure types.

We also observed differences in age at tDCS treatment and disease duration prior to tDCS treatment between responders and non-responders. Age in the responder group ranged from 2 years to 7 years, while that in the non-responder group ranged from 9 to 25 years. Thus, non-responders were older than responders. One recent study estimated the difference in electric fields induced by tDCS between children and adults (32). Under the same stimulation parameters (e.g., current intensity and electrode position), electric field strength in the target cortex were significantly greater in children than in adults. Furthermore, children exhibited reduced skull thickness when compared with adults (32). The skull is much less conductive than other types of tissue, reducing the transmission of current generated by tDCS (33). The relatively thinner skulls of children may allow more current to reach the cortex (32), which may in part explain the better response of younger patients in our study. For the responder group, the disease duration prior to tDCS treatment ranged from 6 months to 3 years, while that for the non-responder group ranged from 7 years to 14 years. The non-responder group had a relatively longer duration of disease prior to tDCS treatment. An accelerating pattern in disease course has been revealed for a subgroup of patients with epilepsy (34). Thus, the activity within epileptic network may differ for patients with epilepsy, with regard to short or long disease duration. This might also in part explain the difference in patients' response to tDCS treatment in our study.

### Repeated Treatment and Current Intensity

Patient 1 was seizure-free for 3 months after one 14-day block of cathodal tDCS treatment. Patient 3 was also seizure-free after two 14-day blocks of tDCS treatment, and this effect lasted for 6 months with repeated treatment blocks. Seizure frequency continued to decrease as the number of treatment blocks increased in Patients 2 and 3, suggesting that the effect of tDCS may be cumulative. Auvichayapat et al. and San-Juan et al. reported that 5 days of tDCS can reduce seizure frequency for 1 month (25) and 2 months (23), respectively. In addition, Tekturk et al. reported that only 3 days of tDCS can reduce seizure frequency for up to 1 month(23). Since our research adopted a longer duration of treatment (14-day block), our findings regarding the therapeutic effect of tDCS are consistent with those of previous studies. However, different tDCS settings may yield non-linear effects (35). And the optimal number of treatment blocks/sessions for controlling epilepsy remains unknown due to a dearth of relevant research. To the best of our knowledge, the present study is the first to adopt a 14-day treatment protocol in patients with epilepsy. The effects of long-term tDCS (e.g., 2–3 weeks) have been extensively studied in patients with chronic pain and depression (14). Further studies are required to determine the effects of long-term/repeated tDCS in patients with epilepsy, as well as the appropriate number of sessions, treatment blocks, and the time course of the observed effects.

In the present study, we utilized 1-mA constant current stimulation for Patient 1 due to her young age (2-year-old), while 2-mA stimulation was used in all other cases. Auvichayapat et al. used 1 mA for patients ranging from 6 to 15 years old, reporting a single adverse event (i.e., erythematous rash under the reference electrode) in the active tDCS group, which resolved within 2 h (20). In another study, Auvichayapat et al. used 2-mA stimulation for 22 pediatric patients ranging from 3 to 9 years old (25). In these studies, the youngest patient with epilepsy who underwent 2-mA tDCS was 3 years old. Based on these literature, Patient 1 in our study underwent 1-mA tDCS, and she remained seizure-free for 3 months. All patients in our study tolerated the treatment well, in accordance with the findings of these previous studies. Nonetheless, further research is required to determine the most appropriate current intensity for tDCS.

## Conclusion

Although our study is limited due to its small sample size and lack of a sham control group, four of the seven included patients exhibited gradual improvement, and antiepileptic effects continued to increase with repeated tDCS treatment. Our results indicate that tDCS is a promising therapeutic method for the treatment of pharmacoresistant ES and further studies are warranted in the future.

## Author Contributions

DY and YL: patient collection and follow-up. QD, ZZ, XiZ, XuZ, and TL: tDCS treatment. LL, LZ, and ZH: ictal symptom classification and EEG analysis. YW: study design.

### Conflict of Interest Statement

The authors declare that the research was conducted in the absence of any commercial or financial relationships that could be construed as a potential conflict of interest.
